# Fusion’s Location and Quality within the Fixated Segment Following Transforaminal Interbody Fusion (TLIF)

**DOI:** 10.3390/healthcare11212814

**Published:** 2023-10-24

**Authors:** Ahmad Essa, Munder Shehade, Oded Rabau, Yossi Smorgick, Yigal Mirovsky, Yoram Anekstein

**Affiliations:** 1 Department of Orthopedics, Shamir (Assaf Harofeh) Medical Center, Zerifin 7033001, Israel; mondershe@clalit.org.il (M.S.); odedr@shamir.gov.il (O.R.); yossis@shamir.gov.il (Y.S.); mirovski@shamir.gov.il (Y.M.); yorama@shamir.gov.il (Y.A.); 2 Faculty of Medicine, Tel Aviv University, Tel Aviv 6997801, Israel; 3 Spine Unit, Department of Orthopedic Surgery, Shamir (Assaf Harofeh) Medical Center, Zerifin 7033001, Israel

**Keywords:** fusion, transforaminal lumbar interbody fusion, posterolateral fusion, degenerative spine, posterior stabilization, intervertebral fusion, Lee criteria, long-term results

## Abstract

Transforaminal interbody fusion (TLIF) has gained increased popularity over recent decades and is being employed as an established surgical treatment for several lumbar spine pathologies, including degenerative spondylosis, spondylolisthesis, infection, tumor and some cases of recurrent disc herniation. Despite the seemingly acceptable fusion rates after TLIF (up to 94%), the literature is still limited regarding the specific location and quality of fusion inside the fixated segment. In this single-institution, retrospective population-based study, we evaluated all post-operative computed tomography (CT) of patients who underwent TLIF surgery at a medium-sized medical center between 2010 and 2020. All CT studies were performed at a minimum of 1 year following the surgery, with a median of 2 years. Each CT study was evaluated for post-operative fusion, specifically in the posterolateral and intervertebral body areas. The fusion’s quality was determined and classified in each area according to Lee’s criteria, as follows: (1) definitive fusion: definitive bony trabecular bridging across the graft host interface; (2) probable fusion: no definitive bony trabecular crossing but with no gap at the graft host interface; (3) possible arthrosis: no bony trabecular crossing with identifiable gap at the graft host interface; (4) definite pseudarthrosis: no traversing trabecular bone with definitive gap. A total of 48 patients were included in this study. The median age was 55.6 years (SD ± 15.4). The median time from surgery to post-operative CT was 2 years (range: 1–10). Full definitive fusion in both posterolateral and intervertebral areas was observed in 48% of patients, and 92% showed definitive fusion in at least one area (either posterolateral or intervertebral body area). When comparing the posterolateral and the intervertebral area fusion rates, a significantly higher definitive fusion rate was observed in the posterolateral area as compared to the intervertebral body area in the long term follow-up (92% vs. 52%, *p* < 0.001). In the multivariable analysis, accounting for several confounding factors, including the number of fixated segments and cage size, the results remained statistically significant (*p* = 0.048). In conclusion, a significantly higher definitive fusion rate at the posterolateral area compared to the intervertebral body area following TLIF surgery was found. Surgeons are encouraged to employ bone augmentation material in the posterolateral area (as the primary site of fusion) when performing TLIF surgery.

## 1. Introduction

Lumbar interbody fusion is a well-established surgical treatment for several lumbar spine pathologies. These include degenerative spondylosis, spondylolisthesis, infection, tumor and some cases of recurrent disc herniation [1,2,3]. Lumbar interbody fusion can be performed by employing several surgical approaches, including posterior, transforaminal, anterior and lateral [4], either by open or minimally invasive techniques [5,6,7,8,9].

Historically, the first lumbar fusion procedure was introduced by Hibbs and Albee et al., employing trans-spinous process fixation and wiring techniques [10,11]. Thereafter, this technique was succeeded by an instrumented posterolateral fusion with facet screws [10,11]. However, due to an increased concern for pseudarthrosis following posterolateral fusion, a new technique involving lumbar interbody fusion was developed by Briggs and Miligan et al. in 1944, rendering an increased fusion rate compared to posterolateral fusion [12].

In recent decades, transforaminal lumbar interbody fusion with posterolateral fusion (TLIF) has gained increased popularity in the surgical treatment of various lumbar degenerative diseases [3,13]. During the surgery, indirect foraminal decompression of the neural elements is achieved, as the intervertebral disc is replaced with an intervertebral implant and bone graft, restoring proper intervertebral space and height, with subsequent posterolateral pedicle fixation for immediate construct stabilization [14,15]. The clinical results of successful fusion are depicted in alleviated post-operative pain scores and regained daily function [16]. As such, Kakadiya et al., reporting data from 120 patients following TLIF surgery, demonstrated approximately 70% pain reduction in the visual analogue scale (VAS) score and over 60% reduction in the Oswestry Disability index (ODI) [16].

TLIF is purported to be superior to the traditional posterolateral fusion (PLF) as it provides additional anterior column stability and 360 degrees fusion [14,17,18,19]. However, there are mixed reports [16,19]. Levin et al., comparing PLF and TLIF procedures for the treatment of spondylolisthesis in a relatively large meta-analysis, demonstrated a significant improvement in terms of ODI favoring TLIF surgery, with pooled estimate effect size of −3.73 (95% confidence interval (CI), −7.09 to −0.38, *p* = 0.03). However, in the same meta-analysis, reporting from one observational study, health-related quality of life (HRQoL) scores were similar for both procedures [19]. Kim et al., evaluating almost 100 patients who underwent either PLF or TLIF procedures, reported similar improvement odds in all patient-reported outcome measures at the 2-year follow-up. Furthermore, similar odds for complications, revision, and quality-adjusted life years (QALY) were noted [20]. Hoy et al., in a randomized clinical trial, through comparing PLF and TLIF in terms of post-operative functional outcomes, showed comparable improvement rates, with no statistical difference in daily function, work, leisure, or anxiety [21]. In another recent extended systematic review, summarizing data from 21 studies with 3686 patients, TLIF and PLF procedures showed similar improvement in terms of patient-reported outcomes [22]. Nevertheless, in reviewing the literature regarding fusion rates following both procedures, a slightly significant difference favoring TLIF surgery is presented [6,19,23]. In a recent meta-analysis comparing TLIF and PLF in terms of fusion rate in patients undergoing fusion for spondylolisthesis, TLIF demonstrated significantly higher fusion rates as compared to PLF [19]. Park et al., comparing fusion rate and its relation to the number of fixated segments after PLF and TLIF employing only local bone graft, showed 50.3% unilateral fusion after PLF compared to a 50.8% fusion rate in at least one area after TLIF at the 12-month follow-up. Moreover, increased fusion rates with the increase in the number of fixated segments were observed [23].

Despite the seemingly acceptable fusion rates after TLIF as compared to PLF (94% versus 84%) [19], the literature is still limited regarding the specific location and quality of fusion inside the fixated segment following TLIF surgery (posterolateral versus intervertebral body area). In a recent study by Rickert et al., evaluating post-operative cage migration and subsidence following TLIF surgery, increased rates of post-operative migration (85%) and subsidence (58%) were noted [24]. However, these findings were not associated with reduced bony fusion. Thus, this reflects the possibility that the primary fusion’s location following TLIF is located in the intervertebral body area [23,24]. In this study, we aim to evaluate the fusion’s quality and its specific location, intervertebral versus posterolateral fusion, following TLIF surgery. We hypothesize that an increased fusion rate in the posterolateral area is present; thus, further bone graft augmentation in the posterolateral area may aid in preserving a higher fusion rate following TLIF surgery.

## 2. Materials and Methods

### 2.1. Study Design

This is an observational single-center retrospective study using a tertiary medium-sized hospital’s registry data between the years 2010 and 2020. This study complies with Strengthening the Reporting of Observational studies in Epidemiology (STROBE) guidelines for observational studies [25].

### 2.2. Study Eligibility

**Figure 1:**
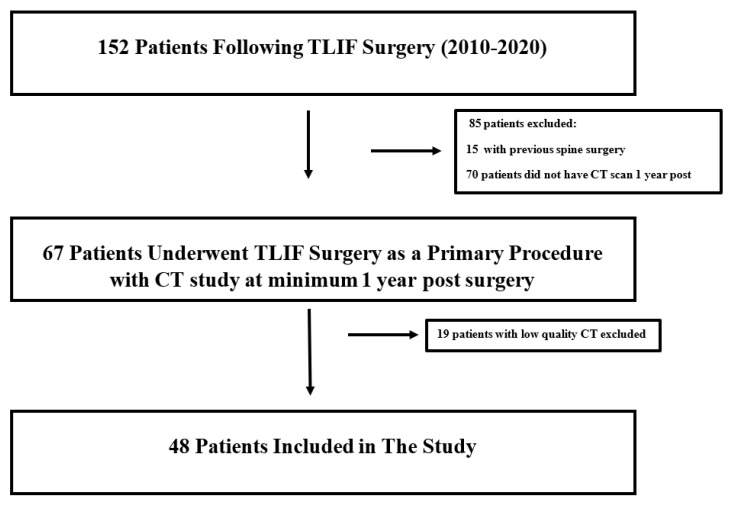
Flow diagram for study cohort creation.

All patients who underwent TLIF surgery between the years 2010 and 2020 with post-operative computed tomography (CT) study at a minimum of 1 year following surgery were identified using specific International Classification of Disease 9th revision (ICD-9) procedure codes. Patients below 18 years of age and/or with previous spine surgery were excluded. Patients with a history of malignancy, radiation exposure, or chemotherapy were excluded. Moreover, low quality CT studies were excluded (Figure 1).

### 2.3. Patients’ Imaging Assessment

A senior orthopedic surgeon was assigned to review and evaluate each CT study. The evaluation process was conducted under close supervision of the head of the spine unit. All included CT studies were performed using high resolution CT scanners and were reviewed using the Sectra PACS (picture archiving and communication system) (v. 23.2.6, 2022, Sweden). All CT studies were performed at a minimum of 1 year following the surgery with a median of 2 years.

### 2.4. Patients’ Exposure

All patients underwent TLIF surgery due to various degenerative spine diseases. Auto local bone graft was employed in all participating patients, in both posterolateral and intervertebral areas. All implants employed in this study consisted of CD Horizon Legacy^TM^ implants and Capstone Peek cages (including sizes; 8 to 12 mm) (Medtronic, Sofamor-Danek, Memphis, TN, USA).

### 2.5. Outcomes

**Figure 2:**
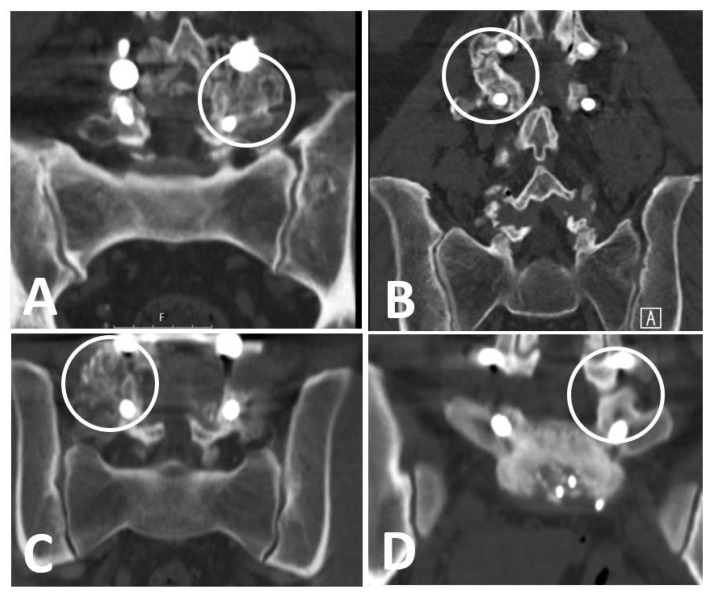
Examples of Lee’s fusion criteria. (**A**) Definite fusion, (**B**) probable fusion, (**C**) possible arthrosis, (**D**) definite pseudoarthrosis.

The primary outcome was the occurrence of fusion at the posterolateral and\or intervertebral area. Fusion’s rate and quality pertaining to each area were evaluated and compared based on Lee’s criteria (Figure 2) [26], as follows:

(1) Definitive fusion: definitive bony trabecular bridging across the graft–host interface.
(2) Probable fusion: no definitive bony trabecular crossing but with no gap at the graft–host interface.
(3) Possible arthrosis: no bony trabecular crossing with identifiable gap at the graft–host interface.
(4) Definite pseudarthrosis: no traversing trabecular bone with definitive gap.

### 2.6. Background Covariates

Background characteristics included: age at the time of the CT (years), sex (male or female), comorbidities (hypertension and diabetes), number of operated segments, specific operated lumbar spine level (L1 to S1), cage size (mm), and post-operative complications (infection, hardware malalignment or migration, and adjacent level disease).

#### Statistical Analysis

Descriptive statistics were calculated for all background characteristics and univariable analysis was conducted using the McNemar test for nominal data. Interval data were analyzed using *t*-test for normally distributed data (Kolmogorov–Smirnoff) or Mann–Whitney U test if not normally distributed. A multivariable model was conducted while controlling for possible confounders, including the number of fixated segments and cage size. A power analysis determined that 45 patients were needed to detect a difference of 40% in fusion rate with 80% power and a significance level of *p* ≤ 0.05. All analyses were performed using the SPSS packages (version 23) (IBM, Chicago, IL, USA). Figures were created using Excel (Microsoft 365 package).

## 3. Results

The initial hospital database search retrieved 152 patients who underwent TLIF surgery between the years 2010 and 2020. After excluding 104 patients who had either previous spine surgery, malignancy, CT scan prior to 1 year post-surgery or low quality CT, a total of 48 patients were included. Additional information is presented in Figure 1.

### 3.1. Patients’ Characteristics

The median age was 55.6 years (SD ± 15.4) with almost 1:1 male to female ratio (26 males versus 22 females). The median time from surgery to post-operative CT was 2 years (range, 1 to 10 years). The main comorbidities included: hypertension in 27% (13/48) of patients and type II diabetes in 25% (12/48) of patients. Thirty three patients (67.3%) underwent one segment fixation, including twenty patients (42%) who underwent fusion at L4–L5, eight patients (17%) at L5–S1, three patients (1%) at L3–L4, one patient at L2–L3 and one patient at L1–L2. Local bone graft was employed in all patients in both posterolateral and intervertebral body areas. No allograft was used in this study. The most common intervertebral body cage size used was 10 mm (52%). In terms of post-operative complications, six patients developed adjacent level disease, three patients had migration or malalignment of hardware and one patient had post-operative infection.

### 3.2. Fusion Rate and Quality

**Figure 3:**
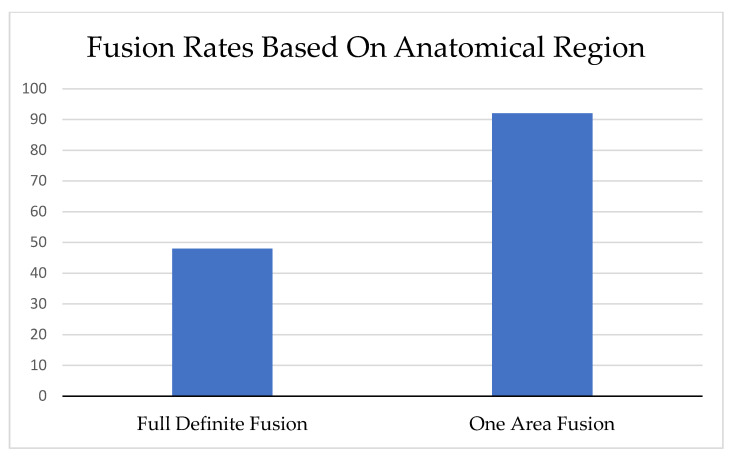
Fusion rates based on anatomical region. Full definite fusion refers to fusion occurring in both posterolateral and intervertebral body area; one area fusion refers to fusion occurring solely either in the posterolateral or intervertebral body area.

Full definitive fusion in both posterolateral and intervertebral body areas was observed in 48% (23/48) of patients, with 92% (44/48) achieving definitive fusion in at least one area (Figure 3). When comparing the fusion rates between posterolateral and intervertebral body areas, a significant higher definitive fusion rate was demonstrated in the posterolateral area compared to the intervertebral body area (92% vs. 52%, *p* < 0.001). Conversely, a higher probable fusion rate was demonstrated in the intervertebral body area compared to the posterolateral area (42% vs. 8%, *p* < 0.001). Additional information is presented in Table 1.

**Figure 4:**
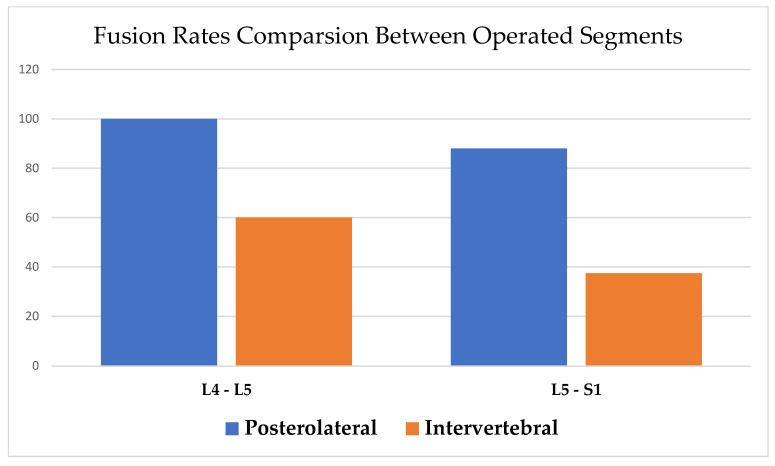
Fusion rate comparison between operated segments.

Stratifying the results pertaining the specific fixated segment showed similar fusion rate trends, with increased definitive fusion rate in the posterolateral area compared to the intervertebral body area (L4–L5: 100% vs. 60%, and L5–S1: 88% vs. 37.5%, *p* < 0.001) (Figure 4). In the multivariable analysis, accounting for the number of fixated segments and cage size, the results remained statistically significant (odds ratio (OR) = 15.9, 95% CI, 1.02 to 247.3, *p* = 0.048) (Table 2).

## 4. Discussion

In this observational study reporting data from 48 patients following TLIF surgery, a significantly increased odds was demonstrated of definite fusion in the posterolateral area compared to the intervertebral body area, regardless of the number of operated segments or cage size. This association remained statistically significant even after stratifying via the specific operated lumbar spine level.

TLIF with posterior stabilization surgery is suggested to be biomechanically more stable than traditional PLF surgery. The advantages of TLIF surgery include additional anterior column stabilization, indirect foraminal decompression, increased lumbar lordosis and 360-degrees of vertebral fusion [14,17,19,27,28,29,30,31,32,33]. In a thorough biomechanical review by Schmoelz et al., combined posterior and intervertebral cage stabilization demonstrated a marked increase in stability in all tested motion planes compared to other construct combinations [29]. A recent meta analysis by Levin et al., pooling data from seven different studies, showed that TLIF in patients treated for spondylolisthesis was associated with a significantly higher possibility of achieving solid fusion at 2 years follow up compared to PLF (94% vs. 84%) [19]. Another systematic review by Dantas et al., comparing data from 12 studies in terms of interbody fusion procedures versus PLF, demonstrated a higher fusion rate in the intervertebral body fusion group compared to PLF (OR = 0.47, 95% CI, 0.26 to 0.86) [28]. Moreover, Challier et al., comparing TLIF and PLF in patients undergoing one level fixation for degenerative spondylolisthesis, showed similar results favoring TLIF in terms of fusion rate (96% vs. 56%) [34]. However, though extensive research and effort has been made in the literature to evaluate the post-operative outcomes of TLIF surgery, very little is known pertaining to the specific fusion’s location and its variation between the different anatomic structures inside the fixated segment following TLIF surgery. A recent study by Rickert et al., evaluating pseudarthrosis and cage position following TLIF, demonstrated that post-operative cage migration and subsidence occurred in 85% and 58% of all patients, respectively. However, no correlation between these findings and reduced bony fusion was noted, implying a possibility of a reduced role for anterior column stability in the overall general construct stability [24]. In this study, a significantly higher definitive fusion rate was observed in the posterolateral area compared to the intervertebral body area following TLIF surgery at a median follow up time of 2 years. Possible confounders, such as the number of fixated segment and cage size, were accounted for in the multivariable analysis with a significant association favoring fusion rate at the posterolateral area.

The findings in this study concur with previous studies suggesting possible low fusion rates at the intervertebral body area following TLIF surgery [23]. Park et al., adapting his own criteria, evaluated the fusion rate using only local bone graft in patients undergoing laminectomy and instrumented fixation with or without intervertebral body support. A bilateral fusion rate of 44.4% following posterolateral fusion was demonstrated. This was compared to a rate of 5.4% in patients with anterior intervertebral body support at 12 months of follow up in single level decompression [23]. However, in the same study when employing less stringent criteria, the observed fusion rates in the intervertebral body support group went up to 43.5%. Moreover, Park et al.’s criteria for intervertebral body fusion required full bilateral fusion at the posterolateral area to be present, thus no specific isolation and comparison between the fused areas inside the fixated segment (posterolateral versus intervertebral body) was conducted. In our study, employing the criteria of Lee et al. [26], a thorough evaluation with specific anatomic location assessment inside the fixated segment was performed, demonstrating significantly higher fusion’s rate and quality in the posterolateral area compared to intervertebral body area, regardless of the specific fixated segment or number of segments operated.

Bone graft augmentation is considered an important surgeon-controlled factor to enhance fusion’s rate during spinal fusion procedures [35,36,37]. Li et al., comparing biomechanical stability in a stimulation model, demonstrated a comparable stability between unilateral pedicle screw fixation and bilateral fixation after complete bone graft fusion [38]. Yoo et al., with the aim of determining the optimal bone graft choice, compared different mixture ratios and volumes of autograft in patients who underwent TLIF surgery, showing a consistent increase in fusion rates with each increase in autograft ratio and\or volume [39]. A recent meta analysis by Tavares et al., pooling data from 64 studies to compare local bone graft, autologous iliac crest bone graft, allograft, and alloplastic bone graft in terms of achieving solid fusion, showed a higher proportion of fusion following the employment of local bone graft (95.3%, CI 89.7 to 98.7) compared to autologous bone graft (88.6%, CI 84.8 to 91.9), allograft (87.8%, CI 80.8 to 93.4), and alloplastic bone graft (85.8%, CI 75.7 to 93.5) [35]. Abou-madawi et al., comparing the use of local autograft and iliac crest bone graft in pedicular screw fixation-augmented TILF, found similar fusion odds (93% versus 94.5%) with no statistical difference between the groups [40]. In a recent throughout systematic review, summarizing data from 22 articles comparing the evidence regarding the employment of various types of bone grafts during TLIF surgery, all employed bone grafts were found to increase the odds of lumbar fusion, with iliac crest bone graft and recombinant human bone morphogenetic protein-2 (rhBMP-2) demonstrating the best results [41]. These results stress the importance of the employment of bone graft during TLIF surgery, such as enhancing fusion rates and the stability of the fused segments. In this study, local bone graft was employed in both posterolateral and intervertebral body areas in all patients, thus minimizing the potential bias of the observed fusion rates based on surgeon’s preference to use, or choice of, bone graft.

A possible theory for the increased fusion rate at the posterolateral area following TLIF surgery could be attributed to better local vascularity and blood supply, as the segmental arteries branching from the aorta pass bilaterally under the pedicles in the posterolateral area of each vertebra providing blood supply from dorsal to ventral. On the other hand, the intervertebral body disc space is considered a relatively avascular zone, with limited blood supply via small arterioles at the vertebra bodies’ endplates. Another possible explanation could be related to the surgical technique in which the cortical bone and other posterior elements in the posterolateral regions is removed, exposing cancellous bone, and prompting the bone healing process with a higher probability for fusion. Meanwhile, in the intervertebral body space, avascular cartilaginous tissue is removed prompting less incentive for the bone healing process and fusion. However, no evidence was found in the literature to support these claims. Moreover, as the stability of the segment treated with the intervertebral cage is closely related to the external compressive preload applied on the cage, as shown by Patwardhan et al. [42], an insufficient external compressive preload exerted in the intervertebral body space may lead to micro motions with subsequent reduced fusion quality and pseudoarthrosis [2,42]. These claims could be supported by a recent study conducted by Xu et al., comparing the specific fusion rates within the intervertebral body area through dividing it to five zones, demonstrating a significant heterogeneity in terms of fusion rates between these zones. For example, the inner (middle) cage zone which faces the maximal load was associated with the highest odds for fusion (95%) followed by the posterior (dorsal) zone (74%) and the anterior (ventral) zone (58%) [43].

Our study has several prominent advantages, including a good study sample with adequate power sample size calculation. Fusion quality and location were determined based on Lee’s criteria employing CT studies only in patients with a minimum follow up of 1 year post-surgery to allow proper time for fusion. A senior orthopedic surgeon was assigned to review each CT study, with close supervision of the head of the spine unit. Moreover, a multivariable model adjusting for possible confounding operation-related factors, including cage size and number of operated segments, was used.

### 4.1. Limitation

The main limitation of this study is related to its retrospective design. This type of study can only demonstrate evidence of association but not causation. Although a multivariable analysis with further stratification was employed, it is still possible that certain undocumented variables could interfere with and confound the results. There was a lack of data regarding several patients’ comorbidities, such as smoking and level of activity, which may possibly confound the results. Furthermore, no patient-reported outcomes or pain severity scores were available for analysis in this study. Thus, very limited conclusions can be drawn regarding the impact of the study’s findings on patients’ daily function. In addition, a possible selection bias may present, as there were missing data regarding the reason for conducting the CT for some of the patients.

### 4.2. Future Research

To further investigate the impact of the current study’s findings, future research should consider evaluating the relation between the difference in fusion rates (posterolateral versus intervertebral body area) and patients’ daily functional outcomes. A prospective study could be designed to assess a cohort of patients undergoing TLIF surgery, with a specific focus on monitoring patients’ reported outcomes. This can be performed through employing advanced imaging modalities to evaluate fusion’s rate and quality in each area inside the fixated segment coupled with clinical and functional assessments questionnaires (ODI, HQRoL, or SF-36). In addition, precise data regarding patients’ comorbidities and other important fusion-related factors should be sought. This approach would provide a holistic perspective on the impact of the differences in fusion rates on patients’ daily function, potentially uncovering valuable insights that could aid surgical decision-making and peri-operative care strategies. Furthermore, considering the dynamic nature of spinal fusion, long-term follow-up is crucial to allow adequate time to fusion and to evaluate the sustained influence on patients’ daily function.

## 5. Conclusions

In conclusion, a significantly higher definitive fusion rate at the posterolateral area compared to the intervertebral body area following TLIF surgery was found. This study should be considered as a benchmark study that allow us to further investigate the reason for the decrease in fusion rate in the intervertebral body area following TLIF and its relevance regarding patients’ reported outcomes measures and clinical scores. Surgeons should consider employing bone augmentation material in the posterolateral area (as the primary site of fusion) when performing TLIF surgery.

## Figures and Tables

**Table 1:** Fusion rates and quality according to Lee’s criteria.

Pseudoarthrosis n (%)	Possible n (%)	Probable n (%)	Definitive n (%)	Region\Lee’s Criteria
-	-	4 (8)	44 (92)	Posterolateral area
-	3 (6)	20 (42)	25 (52)	Intervertebral body area

Abbreviations: n, number.

**Table 2:** Multivariable model for the evaluation of fusion rate while accounting for cage size and number of fixated segments.

95% Confidence Interval	Odds Ratio	
1.02 to 247.3	15.9	Fusion area (PL)

Abbreviations: PL; posterolateral.

## Data Availability

The data presented in this study are available on request from the corresponding author. The data are not publicly available due to privacy and ethical reasons.

## Abbreviation

TLIF	Transforaminal lumbar interbody fusion with posterolateral fusion
CT	Computed tomography
SD	Statistical deviation
PLF	Posterolateral fusion
QALY	Quality-adjusted life year
HQRoL	Health-related quality of life
ODI	Oswestry disability index
PACS	Picture archiving and communication system
MM	Millimeters
OR	Odds ratio
CI	Confidence interval
